# Molecular Regulation of Cotton Fiber Development: A Review

**DOI:** 10.3390/ijms23095004

**Published:** 2022-04-30

**Authors:** Masood Jan, Zhixin Liu, Chenxi Guo, Xuwu Sun

**Affiliations:** 1 State Key Laboratory of Cotton Biology, School of Life Sciences, Henan University, 85 Minglun Street, Kaifeng 475001, China; masoodjan@henu.edu.cn (M.J.); zxlsch2019@163.com (Z.L.); chenxi1445@163.com (C.G.); 2 State Key Laboratory of Crop Stress Adaptation and Improvement, School of Life Sciences, Henan University, 85 Minglun Street, Kaifeng 475001, China; 3 Key Laboratory of Plant Stress Biology, School of Life Sciences, Henan University, 85 Minglun Street, Kaifeng 475001, China

**Keywords:** cotton (*Gossypium* spp.), molecular mechanism, phytohormones, fiber development, transcription factors

## Abstract

Cotton (*Gossypium* spp.) is an economically important natural fiber crop. The quality of cotton fiber has a substantial effect on the quality of cotton textiles. The identification of cotton fiber development-related genes and exploration of their biological functions will not only enhance our understanding of the elongation and developmental mechanisms of cotton fibers but also provide insights that could aid the cultivation of new cotton varieties with improved fiber quality. Cotton fibers are single cells that have been differentiated from the ovule epidermis and serve as a model system for research on single-cell differentiation, growth, and fiber production. Genes and fiber formation mechanisms are examined in this review to shed new light on how important phytohormones, transcription factors, proteins, and genes linked to fiber development work together. Plant hormones, which occur in low quantities, play a critically important role in regulating cotton fiber development. Here, we review recent research that has greatly contributed to our understanding of the roles of different phytohormones in fiber development and regulation. We discuss the mechanisms by which phytohormones regulate the initiation and elongation of fiber cells in cotton, as well as the identification of genes involved in hormone biosynthetic and signaling pathways that regulate the initiation, elongation, and development of cotton fibers.

## 1. Introduction

**Figure 1:**
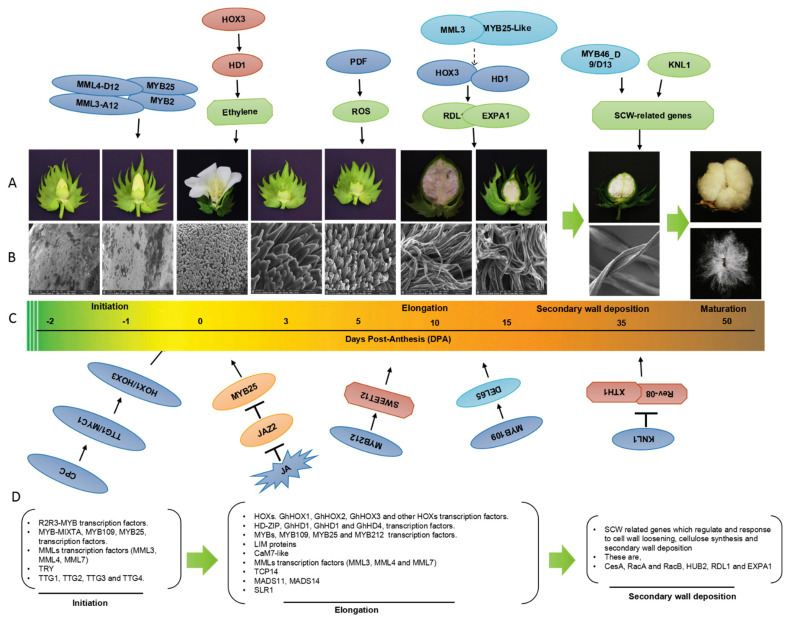
Role of TFs in different stages of cotton fiber development. (**A**,**B**) Boll and fiber development. (**C**) Fiber developmental stages include initiation, elongation, secondary wall biosynthesis, and maturation. (**B**) Scanning electron microscope (SEM) images from -2 DPA (ovule fiber initiation) to 35 DPA (fiber development completion). SEM scale = 100 μm. (**D**) Key TFs are involved in the regulation of cotton fiber development.

Cotton (*Gossypium* spp.) is a globally cultivated, economically important fiber crop. Cotton fibers are one of the main natural resources of the textile industry, and the quality of cotton fibers has a direct effect on the quality of cotton-based textiles [1]. Cotton is also a natural fiber and oil crop that can grow in diverse environments and provide a sustainable source of renewable resources. Upland cotton in particular is known for its high environmental adaptability and its ability to yield large quantities of fiber. Upland cotton has been widely planted, and its output accounts for approximately 95% of all planted cotton [2]. The elongation and development of cotton fibers are complex processes involving multiple genes and pathways. The environment has a substantial effect on the yield and quality of cotton fibers [3]. Because the quality of cotton fiber has a direct effect on the quality of textiles, improving the yield and quality of cotton fiber has long been a major goal of cotton domestication. Sequencing technology has become increasingly used in various scientific fields [4]. The development of mRNA sequencing technology, genome sequencing technology, resequencing technology, and phenotype evaluation methods for cotton has provided new tools for studying the biological mechanisms underlying cotton fiber elongation, and these new approaches could be used by cotton breeders to develop new varieties of upland cotton with improved fiber quality and yield, which would greatly benefit the global textile industry [5]. Transcriptomic analysis permits comparison of the levels of gene expression between cotton varieties at different developmental stages, and this information can be used to enhance fiber production [6,7]. Cotton fibers are single-celled protrusions from the epidermal layer of the ovules; the differentiation and development process can be divided into four overlapping periods: initiation, elongation, secondary cell wall (SCW) biogenesis, and dehydration maturity (Figure 1). The elongation of lint fibers begins at anthesis, whereas the elongation of fuzz fibers begins at 5–10 d post-anthesis (DPA); linear growth continues for approximately 20 d until a length of 25–30 mm is reached [8]. Elongation then ceases, and the secondary deposition of cellulose begins; however, significant overlap between these two processes is often observed [6]. Elongation of the second wave of fibers begins 5–10 d after anthesis, which results in fuzz fibers (referred to as “linters” in the cottonseed trade). These are usually relatively short and stick to the seed when they are ginned [7]. 

Recent studies of the morphology and embryology of cotton ovules have greatly enhanced our understanding of cotton ovule development. Transmission electron microscopy has also been used to study the development of fibers [7,8]. The developmental morphology of cotton flowers and seeds has been examined using scanning electron microscopy (Figure 1B). The formation of cotton ovules and fibers at anthesis has been examined in previous studies (Figure 1A,B). However, not all elements of cotton ovule fiber formation have been discussed. Some previous studies provide detailed descriptions of the formation of early fibers and the surface of the cotton ovule at anthesis through scanning electron microscopy [7,9,10]. In *Gossypium hirsutum*, long lint fibers are produced before or on the day of anthesis (i.e., 0 DPA), although only 15–25% of epidermal cells form commercially usable textile lint fibers. Ovule epidermal cells that start at or after 3 DPA make shorter fibers called linters or fuzz that cannot be separated from seeds by ginning (Figure 1A,B). Fiber mutants in which the growth or development of fiber initials is inhibited are useful tools in phenotypic surveys, as they have shown that the first detectable sign of fiber initiation occurs on the day of anthesis (i.e., 0 DPA). The ratio of fiber initials to total epidermal cells is approximately 25% [9], indicating that fiber output can be enhanced by increasing the number of initials. Breeders have learned that *sucrose synthase* (*SUS*) genes can be used to make cotton fibers that are of better quality [10,11]. The initiation mechanisms of lint and fuzz fibers involve genes such as MYB transcription factors (TFs) [11,12] and phytohormones such as auxin [13,14,15], gibberellic acid (GA) [11,16], brassinosteroid (BR) [17,18], ethylene (ETH) [3,15], abscisic acid (ABA) [10,13], and cytokinin (CK) [13,19]. Cotton fibers, which are used in the textile industry, have long been used to study cell elongation and cell wall biosynthesis [11]. Cotton is one of the world’s most important fiber crop plants and has long been studied. The sequencing of the cotton genome has provided a foundation for transcriptomic studies [11]. These studies have identified several genes (e.g., *MYB25*, *SUS*, *HOX1*, and *HD1*) that play a role in fiber initiation and growth [10]. This information could be used to enhance the quality and production of cotton fiber in various ways. Few papers have reviewed the genes involved in the initiation and growth of cotton fibers, as well as other cellular processes that affect fiber development [4]. 

Here, we aim to provide a complete overview of the key gene families, TFs, and proteins involved in cotton fiber elongation and development that have been identified in previous studies employing omics approaches. The effects of environmental factors (oxidative stressors, notably abiotic stresses) and other variables (phytohormones, fatty acid metabolism, and the actin cytoskeleton) on molecular pathways involved in cotton fiber initiation and elongation have also been examined in previous studies and are summarized in our review [20].

## 2. Transcriptional Regulation of Cotton Fiber Development

**Figure 2:**
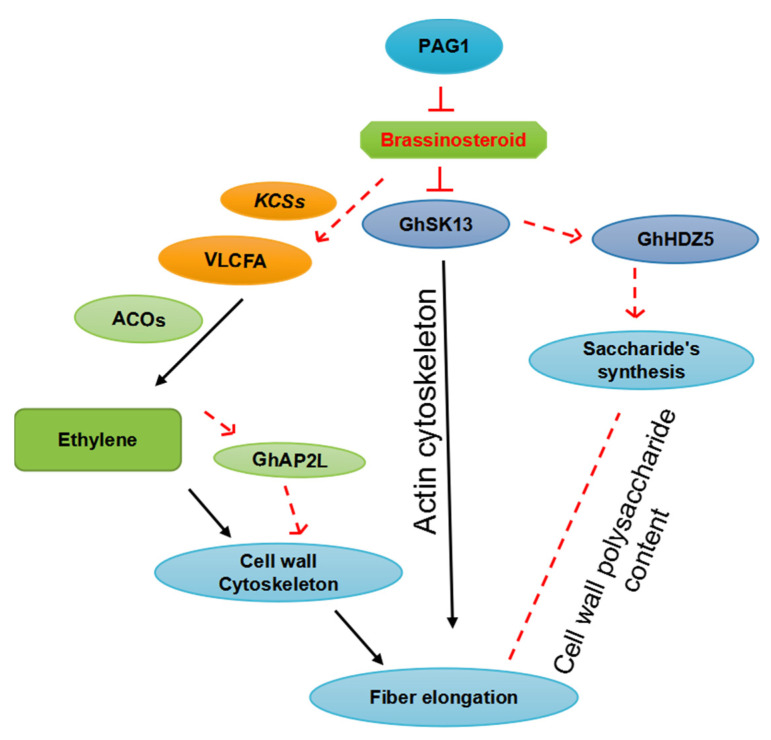
Model representation of how PAG1 and BR regulate fiber elongation. PAG1 catabolism activates endogenous BRs, which stimulate the expression of enzymes involved in very-long-chain fatty acid (VLCFA) biosynthesis, such as KCSs. VLCFAs promote fiber elongation by activating the expression of ETH biosynthesis genes (ACOs). Both ETH and BRs promote fiber elongation by stimulating the expression of cell wall and cytoskeleton-related genes. BRs also affect fiber elongation and *BR-GhSK13* fiber development in cotton.

**Figure 3:**
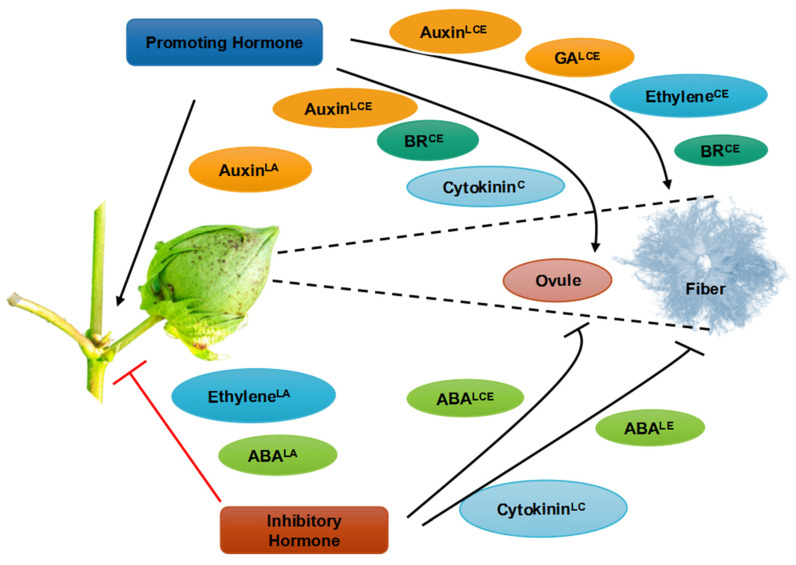
Hormones affecting fiber development, ovule growth, and boll retention. A schematic depiction of a cotton plant’s secondary stem, fruiting stem, and boll (left) and an enlarged view of a fiber-bearing ovule (right) are shown. Hormones that promote boll maintenance, ovule growth, and fiber initiation or growth (left to right) are shown at the top with arrows; inhibitory hormones are shown at the bottom with blocked blunted lines.

**Figure 4:**
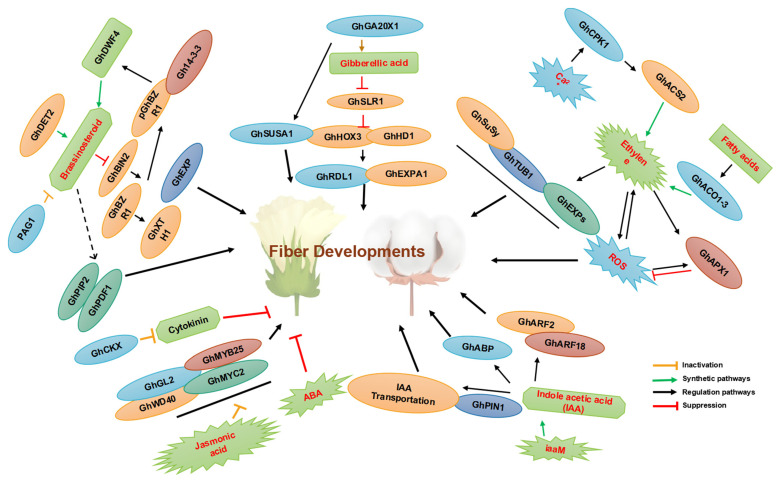
A diagram summarizing the roles of multiple phytohormones in cotton fiber initiation. A schematic model showing the roles of various phytohormones during cotton fiber development. Arrows show promoting actions, and blocked blunted lines show inhibitory actions. Yellow lines indicate the inactivation pathway, green lines indicate the synthetic pathway, black lines indicate the regulatory pathway, and red lines indicate suppression.

Characterization of gene expression profiles has provided new insights into fiber growth processes [21,22]. Tools such as microarrays have been used to identify genes involved in the fiber formation process [7,23]. Several TFs regulate the differentiation and growth of cotton fibers (Figure 1). Mutant cotton lines at different stages of fiber formation are often used to determine the roles that different genes play in fiber development [19]. Comparative transcriptomic studies with fiber mutants have been used to identify key genes or pathways involved in cotton fiber growth and development; for example, *Xu142 fl* has been compared with the wild-type *Xu142* at 0 DPA (days post anthesis) to identify genes involved in lint and fuzz fiber initiation [5,19], and short fiber mutants *LIGON-LINTLESS-1* and *2* (*li1* and *li2*) at 5 DPA have been used to identify the genes involved in lint fiber elongation [7]. RNA sequencing of these short fiber mutants during the fiber elongation stage has identified 531 up-regulated and 652 down-regulated differentially expressed genes (DEGs) in both mutants. The expression of aquaporins is highly down-regulated in both mutants [24]. Subsequent research has shown that only a few genes in *li1* are differently expressed at 1, 3, and 7 DPA; however, a total of 1915 genes are differentially expressed at 5 DPA (984 up-regulated and 931 down-regulated), which corresponds to the peak of fiber elongation [7,9]. At 0 DPA, an Affymetrix cotton GeneChip genome array of isogenic fuzzy lintless (F0) lines with more fuzz and no elongating fibers and normal fuzzy linted (FL) lines of diploid cultivated cotton *Gossypium arboreum* was used. At 10 DPA, the expression of several TFs is down-regulated, including AUX/IAA, AP2-EREBP, bHLH, C2H2, HB, C3H, NAC, MYB, PLATZ, orphans, and WRKY, all of which are involved in hormone signaling pathways, cellular energy metabolism, and fatty acid metabolism [4,10]. All these TFs play a role in lint fiber growth, as well as the down-regulation of genes such as expansins, arabinogalactan proteins, tubulins, and other genes involved in very-long-chain fatty acid synthesis, energy, and cell wall metabolism, which might explain the reduction in fiber growth in F0 [20]. Fatty acid biosynthesis and elongation is the second most up-regulated biochemical pathway during cotton fiber cell elongation, along with three other pathways (Figure 2, Figure 3 and Figure 4) [25,26]. The expression of many cotton genes encoding nonspecific lipid transfer proteins and enzymes involved in various steps of fatty acid chain elongation is highly up-regulated during early fiber development [27], indicating that the biosynthesis of very-long-chain fatty acids or their transport might be required for fiber cell elongation (Figure 2). A comprehensive analysis of lipids has revealed that linolenic (18:3) and palmitic (16:0) acids are the most abundant fatty acids in developing cotton fibers [28]. Omics approaches have been used to identify fiber-elongation-associated genes expressed in the fiber elongation stage in *Gossypium barbadense,* which produces long, fine, and strong fibers compared with *G. hirsutum*. *G. barbadense* has been shown to possess a greater number of expansin and lipid transfer protein genes in the fibers, as well as tubulin, cellulose, and sucrose synthase genes at 22 DPA compared with *G. hirsutum* via expressed sequence tag (EST) pyrosequencing [15,29].

## 3. Genetic Control of Cotton Fiber Quality and Yield

The formation of cotton fibers is a complex process involving several genes acting at various stages (Figure 1). A large number of genes in cotton plants are thought to be involved in fiber formation [30], and only a small number of genes affect genetic diversity in fiber quality across species and cultivars. Hundreds of such loci (also known as quantitative trait loci) have been discovered in tetraploid cotton in recent years. Genome-wide panels of DNA markers have been used to cross parents differing in their fiber properties (such as *G. hirsutum* and *G. barbadense*), and the phenotypes of the offspring have been characterized. Such studies have provided new insights into the chromosomal regions controlling fiber traits [11].

## 4. Role of Plant Growth Regulators in Cotton Growth and Development

Plant growth regulators (PGRs) are non-nutrient organic compounds that affect the physiological processes of plants when applied in small concentrations. These compounds exhibit diverse chemistries and modes of action and can alter crop growth and development in various ways [31,32]. Cotton is a perennial plant with indeterminate growth, and it is responsive to environmental and management changes. PGRs have been used to control growth and enhance yields [33]. Several PGRs are available on the market for cotton production systems. These growth regulators are classified into different groups based on the stages of development at which they trigger a response, including the germination, seedling, vegetative, reproductive development, and harvest stages [31,34]. Few studies have examined their use and mode of action in cotton. They can be applied in-furrow or as seed treatments at planting, during mid-season foliar applications, and late in the season when crops are being prepared for harvest [35]. Some of the advantages of using PGRs for cotton production include their ability to aid the control of vegetative growth, increase yields, improve fiber quality, and facilitate harvesting. PGRs can lead to changes in carbon partitioning, increases in root-to-shoot ratios, increases in photosynthesis, changes in nutrient absorption, improved water status, and enhanced crop canopy shape [31]. The responses to PGRs are ultimately determined by the interaction between heritable characteristics, cultural inputs, and the environment; consequently, the responses of crops to PGRs are not always predictable. Plant mapping techniques have been developed to monitor crop growth and development, and these have been used to characterize the fruiting rates, fruit retention, and distribution of fruit under PGR treatment [36]. Increased boll retention at early fruiting sites enhances crop maturity, which increases the harvesting rate and improves lint quality. PGRs can be used in various ways in cotton production to increase yield and enhance crop management [31,37,38].

## 5. Genes Involved in Fiber Development

Both diploid cotton and tetraploid cotton possess genes that are involved in the production of spinnable fiber. Recent research has shown that *G. hirsutum* has 535 fiber-related genes, including 103 TF genes, 259 fiber development genes, and 173 simple sequence repeat containing ESTs [13,39]. Most TF genes that regulate fiber formation are located in the *Dt* subgenome in tetraploid cotton, and most genes involved in fiber development are found in the *At* subgenome. Polyploidization separates gene functions into different parts of the genome: *Dt*-encoded genes controlling gene expression and *At*-encoded genes controlling the formation of fibers. The stages of cotton fiber development are initiation, elongation, SCW deposition, and maturation. Cotton fiber initiation starts a few days before the opening of flowers. Elongation begins at anthesis for lint fibers or 5–10 DPA for fuzz fibers, and linear growth continues for approximately 20 d, after which the secondary deposition of cellulose begins.

Conserved TFs (MYB, bHLH, and HD-bZIP) have been identified in the regulatory network of fiber development, and the possible mechanisms of action of these TFs have been proposed. The key positive and negative TFs are represented by arrows showing promoting actions and bars showing inhibitory actions. Most of these TFs play a role in fiber (lint and fuzz) initiation and elongation, with the exception of FSN, MYB46_D9/D13, and KNL1, which function in the SCW deposition stage [10]. CPC is a negative factor that functions upstream of TTG1/MYC1. KNL1 is a transcription repressor that inhibits the expression of cell wall and SCW-related genes (Figure 1D).

Table 1 shows the key genes involved in various phases of fiber formation. According to a study that examined 18 genes that play a role in the development of cotton fibers in 22 different wild and cultivated species, 13 full-length homologs of these genes from diploid and tetraploid cotton species possess the same intron/exon splicing positions, but their lengths vary because of insertions and deletions in introns [40].

## 6. Interactions of Phytohormones with Fiber Development

Phytohormones are small endogenous signal molecules in plants [11,13,18]. Plant hormones are regulators of plant growth and development and are also particularly important in the establishment of fiber cells in cotton [13,41]. Gibberellic acid (GA), jasmonic acid (JA), auxin, ethylene (ETH), and brassinosteroid (BR) all contribute to fiber formation, but cytokinin (CK) and abscisic acid (ABA) inhibit fiber growth (Figure 3). Studies of endogenous hormone levels have shown that plant hormones affect the formation of cotton fiber cells [15,42]. 

Plant hormones have been shown to play important roles in the growth of cotton fiber cells and the retention of cotton bolls. Furthermore, measurements of hormone levels have shown that fiber cell initiation and elongation are linked. ESTs from ovules indicate that several phytohormone regulators contribute to the early stages of fiber growth (Figure 3), including auxin, BR, GA, and ABA. The expression of *MIXTA*, *MYB5*, *GL2*, and eight genes in the auxin, BR, GA, and ETH pathways is up-regulated when fiber cells begin to form, but the expression of these genes is down-regulated in the *n1n1* mutant, which is not capable of making fibers. These findings are consistent with the well-known effects of phytohormones on fiber cell development in immature cotton ovules grown in vitro [18,43]. Assembly of ESTs from fiber initials at 1 DPA has revealed several novel genes involved in fiber formation [11]. In addition to *CAPRICE* (*CPC*) genes, many genes that regulate BRs, GTP-mediated signal transduction, cell cycle control, and components of a Ca^2+^-mediated signaling pathway have been identified in fiber at one DPA. This indicates that Ca^2+^ and other signaling pathways play a role in fiber development (Figure 4). It also suggests that the expression of phytohormonal pathway genes is activated before the expression of MYB-like genes is activated, which suggests that phytohormones play a key role in determining cell fate [44,45].

### 6.1. Gibberellic Acid

GA is a key hormone involved in various biological processes in plants, including seed germination, root and stem growth, flower development, fruit ripening, and dormancy [45,46]. Exogenous GA administration enhances fiber elongation in cotton [47], and the application of a chemical that inhibits GA biosynthesis into ovulated culture results in few and short fibers [41]. The concentrations of indole-3-acetic acid (IAA) and ABA, which control fiber elongation during the fiber development stage, are both higher in naturally colored cotton under exogenous GA application. This affects fiber durability, micronaire, and maturation [29,48]. GA accumulation is also related to the elongation of cotton fibers. The number of GA molecules in fiber cells increases and decreases after flowering because GA functions in flower growth and fruit ripening [49]. Endogenous *GA3* is significantly higher in long fiber cotton types than in medium and short fiber cotton varieties [50]. Overexpression of *GA20-OXIDASE1* (*GhGA20ox1*), a key enzyme in GA biosynthesis, results in increased fiber production and the production of longer fibers in transgenic cotton by significantly increasing the content of *GA3* and *GA4* (Figure 4) [50]. Studies of genes that respond to GA have been conducted to determine the function of GA in cell elongation [51]. Transgenic fiber cells overexpressing *GA20 oxidase* show much higher *GhSUSA1* transcript levels than wild-type fiber cells. Furthermore, exogenous bioactive GA promotes *GhSUSA1* transcription in fiber cells and hypocotyls [52]. These findings indicate that GA promotes the formation of SCW in cotton fiber cells by increasing the expression of sucrose synthase genes, which are required for cotton fiber elongation. A mechanism for how GA increases cotton fiber elongation has recently been described. When the GA level is low, one essential regulator in the GA signaling pathway, *GhHOX3*, interacts specifically with the GA suppressor *GhSLR1* and prevents *GhHOX3* from regulating target genes [12]. The interaction between *GhHOX3* and *GhHD1* then enhances the expression of two cell-wall-loosening genes. The HOX3 protein can increase the length of fibers by affecting GA signaling (Figure 4) [49]. 

### 6.2. Jasmonic Acid

The jasmonic acid (JA) signaling pathway is involved in fiber initiation (Figure 1, Figure 2, Figure 3 and Figure 4) [53]. Cotton *GhBLH7-D06* negatively regulates the resistance of cotton to *Verticillium*, and JA can induce the expression of *GhBLH7-D06*. Silencing *GhBLH7-D06* can significantly increase the expression of genes involved in JA biosynthesis and signal transduction genes, such as *GhLOX1-A08*, *GhLOX2-A05*, and *GhLOX3-A09*, and enhance the resistance of cotton to *Verticillium* [44,54]. BLADE-ON-PETIOLE1 (BOP1) is a lateral organ boundary protein, and it can directly activate ATH1 under the action of cofactors to increase the content of JA in plants by promoting the expression of JA biosynthesis genes [39]. According to transcriptomic analysis, the overexpression of a *GhJZA2* inhibitor in the JA signaling system results in reduced fiber initiation and shorter fibers [17], suggesting that the JA signaling pathway plays a key role in fiber elongation. Exogenous GA supplementation significantly increases fiber initiation and elongation. Furthermore, overexpression of *GhJZA2* in *G. hirsutum* cv. *YZ1* also decreases fiber initiation and results in shorter fibers [39]. 

### 6.3. Brassinosteroids

Brassinosteroids (BRs) are a class of polyhydroxylated steroidal phytohormones that play key roles in plant development, growth, and productivity [18]. These hormones regulate the division, elongation, and differentiation of numerous cell types throughout the entire plant life cycle [18,55]. BRs regulate the development of plants via TFs that either repress or induce the expression of downstream genes [11]. BRs play a critical role in the initiation and elongation of cotton fibers. The clearest evidence of the role of BR-induced gene expression in the production of cotton fiber comes from transgenic cotton plants overexpressing a cotton *XTH* called *KC22*. These plants generate fibers that are much longer than those produced by control plants. The synchronized addition of BRZ (a BR inhibitor) and BL (2,4-epibrassinolide) to cultured ovules partly restores fiber development, indicating that BRZ inactivates fiber development through its inhibitory effect on BR production [55]. In vitro treatment of BRs at low doses significantly increases fiber cell elongation; however, inhibiting the production of BRs leads to the inhibition of fiber cell growth [56]. The decrease in steroid levels caused by steroid 5-reductase (DET) is considered a crucial rate-limiting step in the production of BRs. *GhDET2* suppression inhibits fiber cell initiation and elongation, and the seed-coat-specific expression of *GhDET2* increases fiber length (Figure 4) [39]. The exogenous administration of BL increases the elongation of cotton fibers. When flower buds are treated with BRZ, the rate of fiber cell morphogenesis decreases, which indicates that BRs play a role in fiber initiation and elongation. Furthermore, previous studies indicate that *Gh14-3-3* proteins interact with *GhBZR1* to modulate BR signaling, which controls fiber initiation and elongation [57]. *bHLH/HLH* TFs play a key role in fiber formation [58]. However, the specific mechanism by which *bHLH/HLH* TFs regulate BR signaling during fiber formation remains unclear. *GhSK13* appears to be involved in the regulation of saccharide biosynthesis or metabolism, ETH signaling transduction pathway, actin/microtubule-related cytoskeleton, cell wall cytoskeleton status, as well as fatty acid synthesis/metabolism (Figure 4), which eventually affects cotton fiber development and quality. Fiber elongation is significantly suppressed by *PDF1* [59,60]. The expression of these genes is down-regulated in *pag1* (Figure 2), which suggests that BR deficiency affects them. The inhibition of *pag1* fiber elongation stems from the lower expression of *GhPIP2* and *PDF1*. Furthermore, BRs directly or indirectly regulate fiber elongation-related factors such as VLCFA, ETH, the cytoskeleton, and cell-wall-related genes. Thus, BRs might determine the length of the fibers (Figure 2). Cotton fiber quality might be improved by manipulating BR homeostasis. Additional research is required to elucidate the biological activities of TFs in regulating BR signaling at various phases of cotton fiber initiation and elongation [18].

### 6.4. Auxins

Auxins are involved in various developmental processes in plants, including root growth, apical dominance, embryogenesis, vascular differentiation, and the response to internal and external stimuli [13,15]. Previous studies have shown that auxins accumulate in projecting cotton fiber cells, and overexpression of the auxin synthesis gene *iaaM* with an ovule-specific promoter increases the number of fiber initials, which results in a 15% increase in lint output and improved fiber fineness [3]. Auxin response factors (ARFs) are essential components in auxin signaling. ARFs bind to auxin response elements and control the expression of early auxin-responsive genes (*AuxRE*). At the beginning of the fiber formation process, the expression of ARFs from *G. hirsutum* in the *GhARF2* and *GhARF18* subfamilies, as well as six downstream TFs, is high [14,19]. In contrast to *GhARF2* and *GhARF18*, *GhIAA16*, an IAA-induced protein, inhibits the formation of cotton fibers (Figure 3 and Figure 4). The level of *GhIAA16* expression is lower in mutant ovules than in wild-type ovules. However, the number of *GhIAA16* transcripts is high in the *fl* mutant immediately after flowering [9]. Previous studies have shown that the expression of the auxin-binding protein GhABP is up-regulated (approximately 59-fold) in cotton between 0 and 10 DPA. *GhABP* expression has only been observed in elongated fibroblasts, and there is no evidence that *GhABP* is expressed in villous mutants or undifferentiated epidermal cells [61]. Although these findings suggest that *GhABP* plays a role in cotton fiber elongation, the biological function of ABP remains unknown. A comprehensive functional analysis is needed to clarify the biological function of the *GhABP* gene [62]. Several *Rac* genes have been identified in cotton. These genes are highly expressed in fibers and other elongated tissues during fiber elongation, unlike *GhRac1*, *GhRac9*, and *GhRac13*, and their expression decreases with the initiation of SCW biosynthesis [41,62]. The expression pattern of *GhRac1* indicates that it might be involved in regulating the dynamics of the cytoskeleton in elongated fibers and other elongated tissues. *GhRacA* and *GhRacB* are widely expressed in the roots, stems, leaves, hypocotyls, and fiber cells, and the baseline expression of these two genes is highest in fiber cells as well as during elongation [63]. Furthermore, *GhMPK6*, a member of the MAPK family that plays a role in the responses of plants to many biotic and abiotic stresses, has been shown to affect fiber elongation through expression profile analysis. During fiber elongation, the phosphorylation level of *GhMPK6* remains high, and phytohormones increase the phosphorylation level of *GhMPK6* in the fibers [62,64].

### 6.5. Ethylene

Ethylene (ETH) plays various roles in plant growth and development, as well as in the responses of plants to biotic and abiotic stresses. ETH is known for its role in fruit ripening and organ abscission [11,44]. In cotton, ETH is thought to play a role in fiber cell initiation and growth, as shown by the overexpression of *ACO* genes during fiber elongation [65]. Furthermore, exogenous ETH stimulates fiber cell elongation. The expression of *SUS*, *TUBULIN*, and expansin genes is down-regulated in the Xu142 *fl* mutant, and down-regulation of the expression of these genes is required for cell wall formation, the cytoskeleton, and cell wall loosening (Figure 3 and Figure 4) [20]. ETH might promote cell elongation by modulating the expression of sucrose synthase, tubulin, and elongation-related proteins [29,66]. Although upland cotton (*G. hirsutum*) is the most common type of natural fiber, little is known about the regulation of fiber elongation. ETH production is a key biological process during fiber elongation according to the sequencing of a cotton fiber cDNA library and microarray analysis [29,67,68]. The expression of the key ETH-producing genes *1-aminocyclopropane-1-carboxylic acid oxidase1–3* (*ACO1–3*) is higher throughout this developmental phase compared with other developmental phases. The amount of ETH emitted by grown ovules is related to *ACO* expression and the rate of fiber development (Figure 3 and Figure 4) [20]. 

### 6.6. Cytokinin and Abscisic Acid

Levels of abscisic acid (ABA) and cytokinin (CK) are high in the ovules and developing fibers of a *Ligon lintless* (*li*) mutant line with short lint and normal fuzz, and this has been shown to inhibit fiber elongation and fiber initiation, respectively, in an in vitro ovule culture system [16,69], indicating that both hormones have antagonistic effects on cotton fiber development. ABA levels are also higher in the early stages of fiber formation in the *Xu142 fl* mutant [69]. Endogenous ABA levels in cotton ovules are positively linked to short fiber production. The deposition of ABA in 0 DPA ovules is significantly increased in the short fiber mutant *Ligon lintless 2* (*li2*) compared with wild-type plants [70], indicating that ABA is a negative regulator of cotton fiber initiation and elongation. When the *GhCKX* gene is silenced with RNAi technology, the number of seeds increases significantly, and the fiber yield increases slightly, which indicates that CKs are needed for seed development and have an indirect effect on fiber yield (Figure 3 and Figure 4) [71].

## 7. Role of Other Factors in Fiber Development

**Figure 5:**
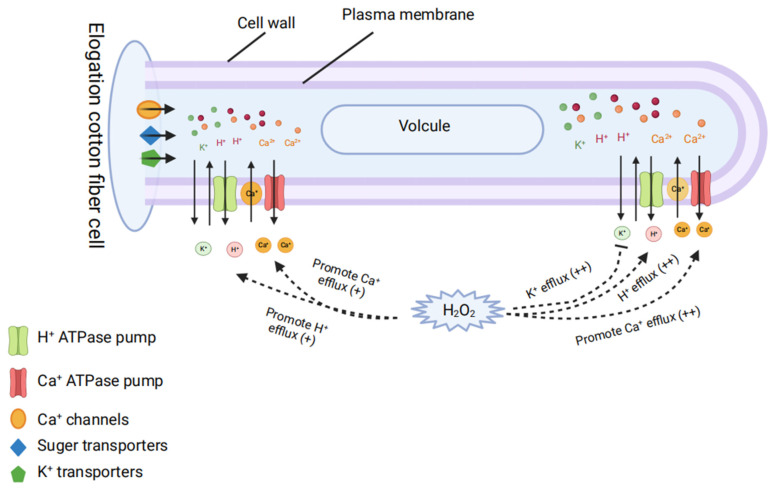
A schematic model of how cotton fiber cells grow, with Ca^2+^, K^+^, and H^+^ channels and ion efflux. Ca^2+^, K^+^, and H^+^ channels and efflux fluctuations are observed in both the tip and the base of the elongating fiber cells that were treated with H_2_O_2_.

Signaling molecules such as calcium ions (Ca^2+^) and reactive oxygen species (ROS) (e.g., H_2_O_2_) play important roles in the growth of cotton fibers. Many different types of Ca^2+^, K^+^, and H^+^ channels and efflux systems are present on the surface of the cell membrane, and they all work together to keep levels of Ca^2+^, K^+^, and H^+^ in cells stable (Figure 5) [72]. Cotton fibers can develop if sufficient Ca^2+^ channels and efflux systems are functioning, suggesting that these transporters might play a role in cotton fiber elongation [54,72]. Both factors are discussed below. The net Ca^2+^, K^+^, and H^+^ effluxes in both areas increases dramatically when cells are exposed to H_2_O_2_. Non-growing cells (20 DPA) show no response to H_2_O_2_ treatment and lack Ca^2+^ and H^+^ oscillations, suggesting that the desensitization of fiber cells and the loss of their ability to respond to H_2_O_2_ might be causally related to the termination of cotton fiber elongation [72].

Nearly all plant cells produce ROS through metabolic processes. The most well-known function of ROS is as a signaling molecule under biotic and abiotic stress. ROS not only play an important role in the formation and elongation of cotton fibers, but they might also act as signaling molecules in responses to various types of abiotic and biotic stress [68]. DEGs involved in H_2_O_2_ homeostasis have been identified during the fiber formation stage, indicating that ROS might play a role in fiber growth [73]. The existence of ROS during cotton fiber initiation has been established via fluorescence imaging of the ROS indicator 2p,7p-dichlorodihydrofluorossein diacetate [74], (Figure 1, Figure 2, Figure 3 and Figure 4). Fiber development is induced in *G. hirsutum* fiber formation mutants treated with H_2_O_2_ [75]. These findings indicate that ROS play a key role in cotton fiber initiation. The *GhAPX1* gene, which encodes an enzyme involved in H_2_O_2_ homeostasis, is expressed at a low level in fuzzless-lintless mutant ovules but at a high level in wild-type fibers at 5 DPA. Treatment of cotton ovules with exogenous H_2_O_2_ and ETH in vitro significantly enhances the activity of ascorbate peroxidase, which promotes fiber cell elongation via the induction of *GhAPX1* expression [76].

Ca^2+^ signaling, which is important for plant growth and development, plays a key role in the elongation of cotyledon fibers (Figure 4) [38,72]. Additionally, silencing of the *GhCaM7*-like gene might result in shortened fibers [77]. Overexpression of *GhCaM7*, a calcium sensor, in fiber cells increases ROS concentrations relative to those of wild-type plants, but silencing of this gene results in a decrease in ROS accumulation [38]. Cotton ovule cultures with no fiber elongation are suppressed in the absence of Ca^2+^ [78]. Additionally, a low Ca^2+^ concentration favors the initiation of fiber production, early elongation, and expression of genes [72]. Calmodulin is often a major component of Ca^2+^ signaling pathways, and the calmodulin inhibitor trifluoperazine slows fiber elongation in the lab at low calcium concentrations [11,78]. The Ca^2+^ signaling genes *CALCINEURIN B-LIKE* (*CBL*) *interacting protein kinase* (*GhCIPK1*), *Calmodulin* (*GhCaM*), and *Glutamate decarboxylase* (*GhGAD*) are expressed at different times [79,80]. 

## 8. Challenges, Conclusions, and Future Directions

More detailed information regarding the genetic and epigenetic factors, as well as phytohormones, would greatly aid our understanding of the initiation and determination of fiber cell fate. This information would also facilitate the manipulation of cotton fiber cell initiation and fate-determining genetic and epigenetic factors using genetic or genome engineering tools, which could increase the number of lint-fiber-producing committed cells on the ovule epidermal surface and promote enhanced yield and quality of the fibers produced. Fiber genomics has helped identify fiber initiation and development-related genes and other regulatory factors. Gene expression analysis enhances our understanding of genetic infrastructure by characterizing the relationship between genotype and phenotype. Additional transcriptomic and proteomic studies are needed to clarify the regulatory mechanisms underlying fiber growth. Previous studies have indicated that several genes and gene families play a role in the growth of cotton fibers. Hormones that affect plant growth and development, such as GA, BR, auxin, CK, ETH, and JA, have been shown to contribute to cotton fiber formation. However, there are still several outstanding problems requiring clarification. For example, the processes that most strongly affect the quality of cotton, as well as how these processes interact, need to be determined. The number of fibers in cotton might be increased and fiber elongation achieved through the exogenous injection of these hormones or the overexpression of associated genes. However, whether some critical components in these pathways contribute to the fiber growth process remains unclear. More functional research on mutant lines is needed to address these knowledge gaps. Several key genes from various pathways that regulate the process of fiber initiation and development in cotton have been identified. There is also a need to evaluate the contributions of these pathways and their interactions to enhancing cotton fiber quality. Next-generation cotton germplasm could be produced more quickly and with higher quality to meet increases in demand. 

## Figures and Tables

**Table 1:** The role of genes involves different stages of fiber development.

Gene Name	Gene Family	Function Stage of Fiber Development
*CIPK1*	CBL-interacting protein kinase	Expressed during fiber elongation
*Exp1*	a-Expansion 1	Positive role in secondary cell wall deposition
*ACT1*	Actin1	Expressed during fiber elongation
*BG*	b-1,4-Glucanase	Positive role in secondary cell wall deposition
*ManA2*	b-Mannosidase,	Expressed during fiber development in elongation
*Pel*	Pectate lyase	Degradation of de-esterified pectin and has a role in fiber elongation
*POD2*	Bacterial-induced peroxidase	Expressed during fiber initiation and elongation
*RacA*	Small GTPase	Expressed during fiber elongation
*RacB*	Small GTPase	Expressed during secondary cell wall thickening
*Sus1*	Sucrose synthase	Expressed in fiber initiation and elongation
*LTP3*	Lipid transfer protein gene	Cutin synthesis during fiber primary cell wall synthesis
*14-3-3L*	14-3-3	Expressed at early stages of fiber elongation
*CelA1*	Cellulose synthase	Expressed in secondary cell wall synthesis, involved in the synthesis of cellulose
*CelA3*	Cellulose synthase	Involved in cellulose biosynthesis stage in developing cotton fibers
*MYB109*	MYB	Positive in initiation and elongation
*MYB25*	MYB	Positive role fiber initiation
*MML3-A12*	MYB	Positive in fuzz fiber initiation
*MML4-D12*	MYB	Positive in lint fiber initiation
*MYB212*	MYB	Positive in elongation
*MYB46-D13*	MYB	Positive role in secondary cell wall deposition
*MYB46-D9*	MYB	Positive role in secondary cell wall deposition
*CPC*	MYB	Expressed during fiber initiation and negative role in the initiation
*TRY*	MYB	Expressed during fiber initiation and negative role in the initiation
*HD1*	HD-ZIP IV	Positive in initiation
*HOX3*	HD-ZIP IV	Positive in initiation
*TTG1*	WD repeat	Positive in initiation
*TTG2*	WD repeat	Positive in initiation
*TTG3*	WD repeat	Positive in initiation
*TTG4*	WD repeat	Positive in initiation
*SLR1*	DELLA	Expressed during fiber elongation and negative role in elongation
*BZR1*	BES1_N	Positive in initiation
*MADS11*	MADS-box	Positive in elongation
*MADS14*	MADS-box	Expressed during fiber elongation and negative role in elongation
*TCP14*	TCP	Positive in initiation and elongation
*JAZ2*	JASMONATE-ZIN-DOMAIN	Negative in lint and fuzz fiber initiation

## Data Availability

All data supporting the findings of this study are available within the paper and online.
